# Effects of incubation temperature on development, morphology, and thermal physiology of the emerging Neotropical lizard model organism *Tropidurus torquatus*

**DOI:** 10.1038/s41598-022-21450-7

**Published:** 2022-10-13

**Authors:** Anderson Kennedy Soares De-Lima, Carlos Henke de Oliveira, Aline Pic-Taylor, Julia Klaczko

**Affiliations:** 1 Laboratory of Comparative Vertebrate Anatomy, Department of Physiological Sciences, Institute of Biological Sciences, University of Brasília, Brasília, DF 70910-900 Brazil; 2 Graduate Program in Zoology, Institute of Biological Sciences, University of Brasília, Brasília, DF 70910-900 Brazil; 3 Laboratory of Applied Ecology, Department of Ecology, Institute of Biological Sciences, University of Brasília, Brasília, DF 70910-900 Brazil; 4 Laboratory of Embryology and Developmental Biology, Department of Genetics and Morphology, Institute of Biological Sciences, University of Brasília, Brasília, DF 70910-900 Brazil; 5 Department of Life Sciences, Natural History Museum, London, SW7 5BD UK

**Keywords:** Physiology, Zoology

## Abstract

Incubation temperature is among the main phenotypic trait variation drivers studied since the developmental trajectory of oviparous animals is directly affected by environmental conditions. In the last decades, global warming predictions have aroused interest in understanding its impacts on biodiversity. It is predicted that the effects of direct warming will be exacerbated by other anthropogenic factors, such as microclimatic edge effects. Although the Brazilian Cerrado biome is one of the most affected by these issues, little is known about the aforementioned effects on its biodiversity. Therefore, the aim of our study is to investigate the influence of incubation temperature on developmental parameters, morphology and thermal physiology traits of the collared lizard (*Tropidurus torquatus*). Furthermore, we discuss our findings regarding lizard developmental biology and the climate change paradigm. Therefore, we incubated *T. torquatus* eggs under five temperature regimes ranging from artificial nest temperature (28.7 °C) to 35.0 °C. We found that elevated incubation temperatures affect several investigated traits: egg mass gain is positively affected, without any influence in newborn mass; incubation period is broadly reduced with temperature increase; survival rate is negatively affected by temperature, constant 35.0 °C regime is confirmed as a lethal incubation temperature, and the sex ratio is affected at 30.0 °C, with a prevailing outbreak of females. Increased incubation temperature also affects body and head size but has no effect on limb size. Newborn thermoregulation and the critical thermal maximum (CT_max_) are not affected by incubation temperature. On the other hand, basal body temperature (T_bb_) and the critical thermal minimum (CT_min_) were positively affected. Thermal physiology was also affected by age, with newborns differing from adults for all analyzed thermal traits. Our findings indicate that future modifications in incubation temperature regimes at nesting sites caused by warming may affect several features of the development, morphology, and thermal physiology of newborns of this species. Laboratory experiments have pointed to possible drastic effects of warming on lizard survival rates, also affecting aspects of its natural history and population distribution. Moreover, in addition to being more vulnerable than adults in aspects such as predation and feeding, *T. torquatus* newborns are also more vulnerable regarding thermal physiological traits.

## Introduction

Developmental pathways are determined by a broad number of factors acting on biological processes during embryogenesis, such as incubation temperature^1^. This factor, often recognized as a strong inducer of phenotypic plasticity under fluctuating environmental conditions, can modulate the phenotype in response to oscillations in metabolic rate and genetic expression^2,3^. Moreover, under conditions of extreme incubation temperature variation, the collapse of ontogenetic processes leads to a non-viable phenotype, thereby interrupting the developmental process^4^.

Some groups of metazoans, such as oviparous reptiles, are especially more susceptible to temperature variations during development^5^. In these heliothermic vertebrates, egg incubation is directly affected by fluctuating conditions in the laying environment and is more pronounced than in species whose embryonic development occurs inside the maternal body^6,7^. Although temperature-mediated phenotypic plasticity is a natural phenomenon, little is known about the possible effects of climate change on biodiversity regarding the increased average temperatures and extreme climate events potentialized by anthropogenic actions^8–11^.

In the context of global climate change, particularly pertaining to the increase in the average global temperature observed in the last decades^12^ and predicted for the next century^13,14^, heliothermic vertebrates, mainly oviparous, have aroused relevant research interest given their vulnerability to such environmental changes^15–20^. Forecasting models based on thermal performance curve studies have shown that lizard susceptibility to the ever-increasing global warming will be enhanced through changes in the foraging period, leading to reduction of energy gain, as well as changes in the potential distribution areas of these species^21,22^. In addition, nest temperature modifications due to higher average surrounding environment temperatures pose direct risks to oviparous species in several ways, such as: reducing birth rates; changing the population sex ratio and posing phenotypic restrictions with ecological implications (i.e. morphological malformations, reduced locomotor performance, altered thermal physiology, among others)^23–30^. In addition to climate change, habitat disturbance has threatened lizard species worldwide^31,32^, especially in biomes where natural landscapes have given up space to establish agricultural fields, as is the case in the Brazilian Cerrado^33–35^. This unfortunate combination has significantly altered abiotic factors—i.e. a shorter dry season and reduced relative humidity^33,36^—and enhanced the microclimatic edge effects in natural environments^32,33^, with little still known about the effects on the highly seasonality-adapted biota of the Cerrado biome^37–40^.

Despite representing one of the largest vertebrate radiations, few squamate reptile species (lizards, snakes and amphisbaenians—so called “worm lizards”) have had phenotypic traits investigated from the incubation temperature perspective^41^. Furthermore, even fewer studies have investigated the effects of egg incubation temperature on thermal physiology features of ecological importance^24^. Finally, none of these studies included Neotropical species, leaving a knowledge gap about the effects that drastic phenological changes can have on these organisms. Herein, we investigate the effect of incubation temperature on egg development, neonatal morphology and the thermal physiological traits of the tropidurid lizard *Tropidurus torquatus*, a medium-size lizard widely distributed throughout South America and extensively studied in the most diverse biological aspects^42–45^. In addition, *T. torquatus* has considerably simplified husbandry and captive maintenance in addition to being one of the few South American lizard species to have a complete post-ovipositional embryonic staging table^44^. The aims of our study are to (i) test the effect of different incubation temperatures on developmental, morphological and thermal physiological traits, and (ii) investigate any differences in thermal physiological traits between neonates and adults of this species.

## Results

The incubation of 144 randomized eggs in five different treatments—(i) artificial nest built in the field (mean temperature of 28.7 °C), (ii) 30.0 °C, (iii) 32.5 °C, (iv) 32.5 °C + 39.0 °C/2 h/day, and (v) 35.0 °C—resulted in the birth of 82 neonates, measured to obtain the morphology and thermal physiology datasets. We also obtained developmental data from the measurement of eggs during the incubation period and thermal physiology data from 37 *Tropidurus torquatus* adults. The developmental traits, morphology and thermal physiology showed significant variation between incubation temperatures, as well as a significant difference in the thermal physiology between neonates and adults, demonstrating that both incubation temperature and age interfere in these traits.

### Influence of incubation temperature on egg mass and volume

**Figure 1:**
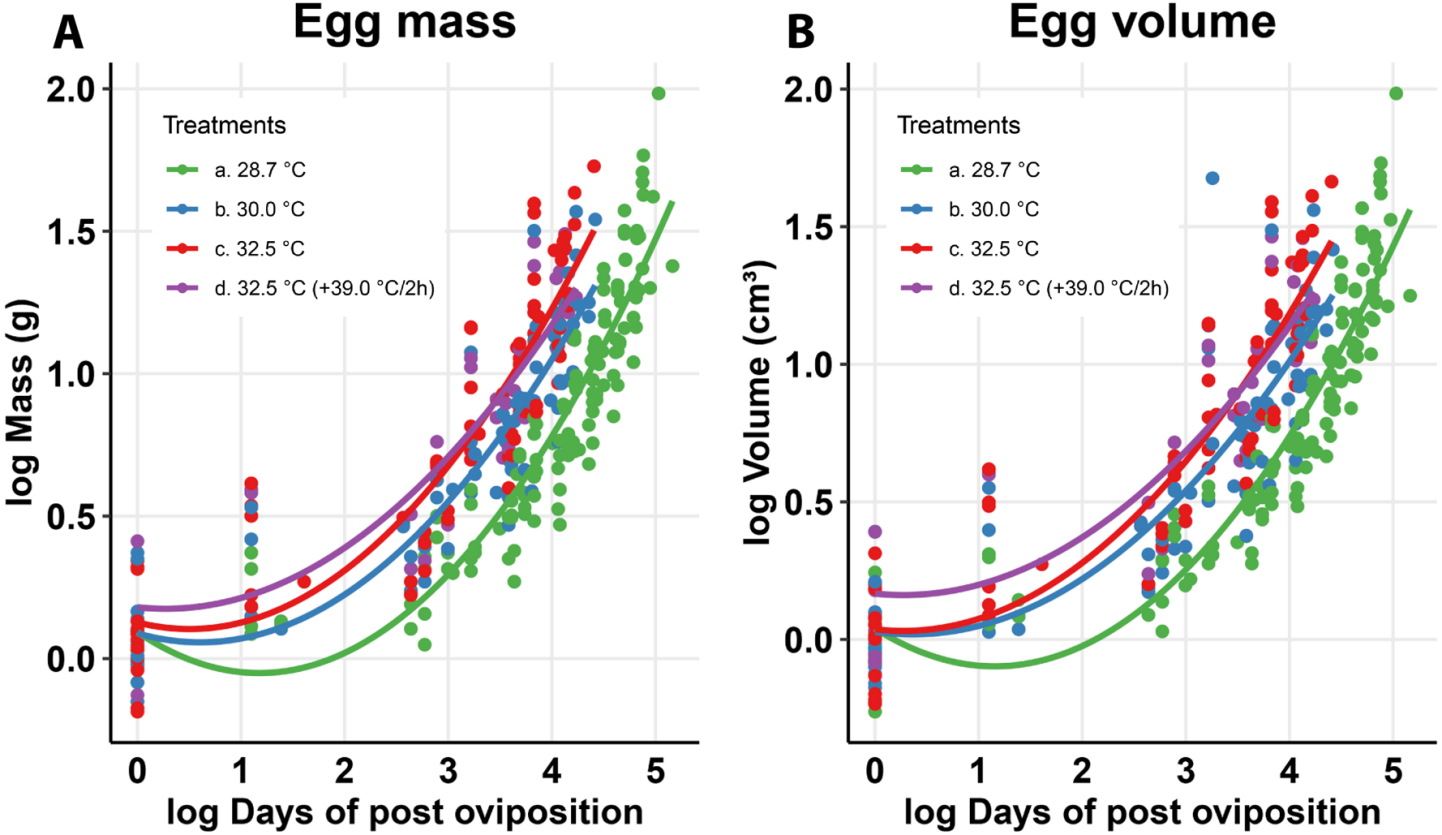
(**A**) Mass and (**B**) volume variation during the incubation of *Tropidurus torquatus* eggs under four incubation temperature regimes.

**Figure 2:**
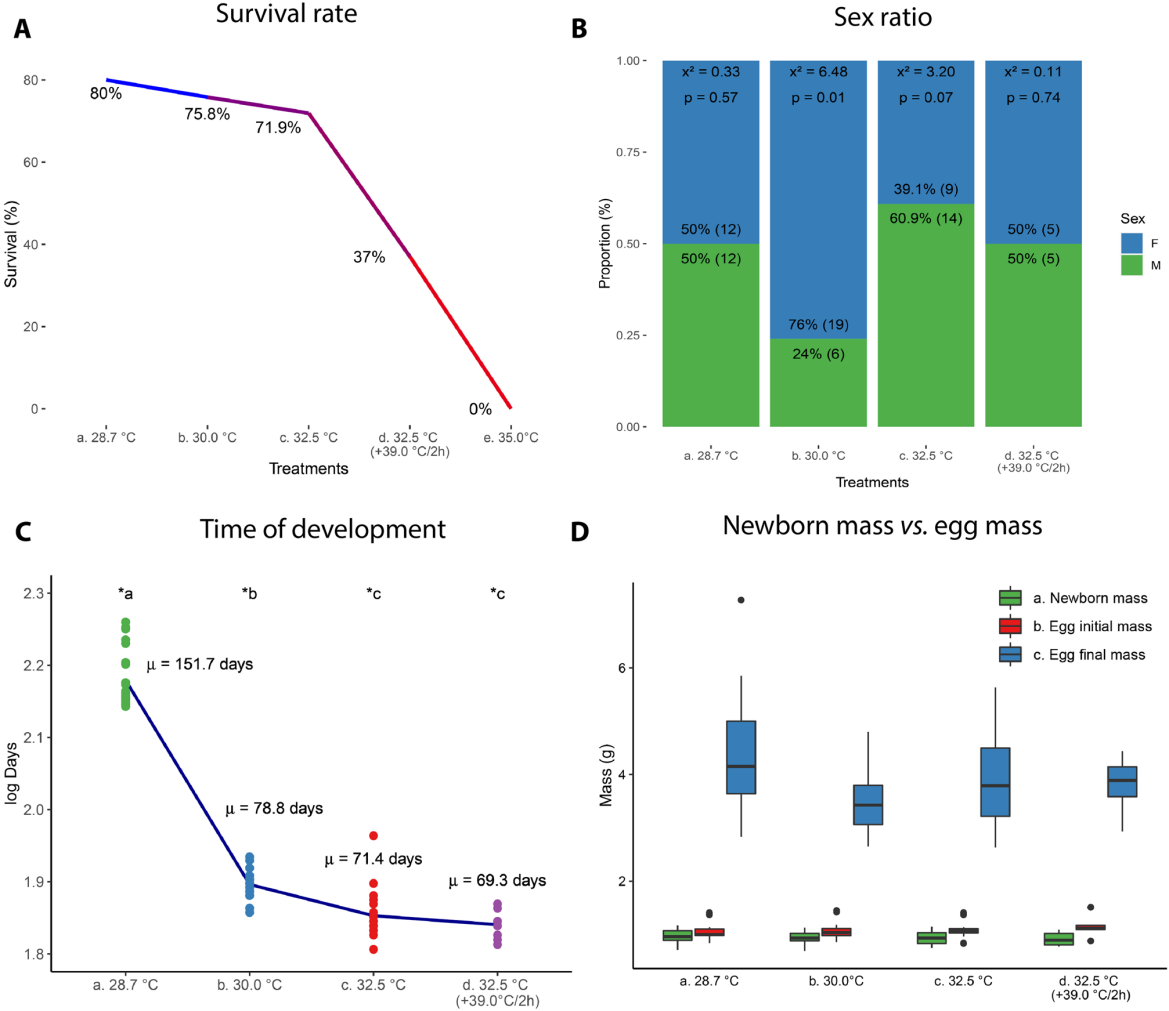
Incubation temperature effect on: (**A**) survival rate; (**B**) sex ratio; (**C**) development time, and (**D**) egg mass of *Tropidurus torquatus* under four incubation temperature regimes.

Egg mass and volume were positively affected by incubation temperature. In both cases, mass and volume did not differ significantly between 28.7 (artificial nest) and 30.0 °C treatments. Artificial nest treatment differed significantly from 32.5 to 32.5 °C (± 39 °C/2 h) treatments. In turn, the treatment 30.0 °C differed significantly from 32.5 °C, but not from 32.5 °C (± 39 °C/2 h). The mean mass and volume values did not differ significantly between eggs incubated at 32.5 and 32.5 °C (± 39.0 °C/2 h) (Fig. 1; Tables 1 and 2). Egg mass increased approximately four times during the incubation period; however, the egg mass gained did not influence newborn mass (Fig. 2.D; ANCOVA, F = 1468.01, *P* < 0.0001; initial egg mass = 1.1 g ± 0.2, final egg mass = 3.9 g ± 0.9, newborn mass = 0.9 g ± 0.1).

**Table 1:** Mass gain rate during the incubation period of *Tropidurus torquatus* eggs under four temperature regimes.

Temperature (°C)	Mean (± sd)		Mass gain rate		Difference in mass gain curve among temperatures			Difference in mass among temperatures		
			b	r^2^	F statistics		*P*	F statistics		*P*
28.7	2.44 (± 1.2)	*a	0.27	0.69	0.4657	ns	0.70	11.892	*	< 0.0001
30.0	2.18 (± 0.9)	*ac	0.25	0.75						
32.5	2.36 (± 1.2)	*b	0.28	0.77						
32.5 (+ 39.0/2 h)	2.40 (± 1.0)	*bc	0.25	0.72						

Significant effect of incubation temperature is indicated by (*); letters *a, *b, & *c denotes the post-hoc difference among treatments; b, ANCOVA slope; F, ANCOVA result; *ns* not significant, *P*, ANCOVA significance; r^2^, coefficient of model adjust; *sd* standard deviation.

**Table 2:** Volume gain rate during incubation of *Tropidurus torquatus* eggs under four incubation temperature regimes.

Temperature (°C)	Mean (± sd)		Volume gain rate		Difference in volume gain curve among temperatures			Difference in volume among temperatures		
			b	r^2^	F statistics		*P*	F statistics		*P*
28.7	2.34 (± 1.2)	*a	0.27	0.70	0.7999	ns	0.49	11.996	*	< 0.0001
30.0	2.12 (± 1.0)	*ac	0.25	0.71						
32.5	2.26 (± 1.2)	*b	0.29	0.78						
32.5 (+ 39.0/2 h)	2.33 (± 1.0)	*bc	0.24	0.68						

Significant effect of incubation temperature is indicated by (*); letters *a, *b, & *c denotes the post-hoc difference among treatments; b, ANCOVA slope; F, ANCOVA result; *ns* not significant; *P*, ANCOVA significance; r^2^, coefficient of model adjust; *sd* standard deviation.

### Influence of incubation temperature on survival rate, development time, sex ratio and morphology

Incubation temperature affected survival rate among treatments: artificial nest temperature, 30.0 and 32.5 °C produced high newborn rates of 80.0%, 75.8% and 71.9% survival, respectively. The 32.5 °C (± 39 °C/2 h) treatment resulted in a 37% survival rate and the 35.0 °C treatment was lethal, without hatching (Fig. 2.A; X^2^ = 28.19, *P* < 0.0001). The sex ratio was only affected at 30.0 °C treatment, resulting in 76% females. Nevertheless, at the 32.5 °C treatment, we registered a ~ 4:6 (female:male) proportion, thus differing from the 1:1 (female:male) proportion registered in the artificial nest and 32.5 °C (± 39 °C/2 h) treatments (Fig. 2B). Incubation temperature also affected development time, with laboratory treatments resulting in approximately half of the incubation period observed in the artificial nest treatment (Fig. 2C).

Morphological variables were affected by incubation temperature (MANCOVA, Pillai = 1.05, F = 2.46, *P* < 0.0001), especially body trunk, tail, and head traits (Table 3). Body length was the only morphological trait affected by sex as a covariate at artificial nest treatment, with males presenting shorter bodies than females (13.6 mm and 14.4 mm, respectively; ANCOVA, F = 5.129, *P* = 0.034). Incubation temperature also influenced tail length, total length, body length, head length, and head height traits. Mass, snout-vent length, interbrachial-nasal length, head width, and limb traits were not significantly affected (Table 3, Supplementary Fig. SF2.A–N).

**Table 3:** Effects of incubation temperature on development time and morphological traits of *Tropidurus torquatus* newborns under four temperature regimes.

Trait	Incubation temperature (°C)				Effects					
	28.7 (n = 24)	30.0 (n = 25)	32.5 (n = 19)	32.5 (+ 39.0/2 h) (n = 14)	Incubation temperature			Sex		
					F statistics		*p*	F statistics		*p*
TOD	151.75 (± 14.0) *a	78.8 (± 3.2) *b	71.43 (± 5.5) *c	69.3 (± 2.8) *c	639.9	*	< 0.0001	0.003	ns	0.96
MAS	0.96 (± 0.1)	0.94 (± 0.1)	0.93 (± 0.1)	0.9 (± 0.1)	0.391	ns	0.76	0.074	ns	0.79
SVL	30.2 (± 1.2)	30.5 (± 1.4)	30.8 (± 1.6)	30.4 (± 1.6)	0.533	ns	0.66	0.048	ns	0.83
TLL	41.5 (± 6.1) *ab	42.0 (± 5.5) *ab	44.6 (± 4.1) *a	39.3 (± 7.5) *b	3.497	*	0.02	0.108	ns	0.74
TTL	71.7 (± 7.1) *b	72.5 (± 6.5) *ab	75.3 (± 5.3) *a	69.7 (± 8.7) *b	4.265	*	0.008	0.161	ns	0.69
BL	14.0 (± 1.0) *b **m < f	14.8 (± 0.9) *a	14.7 (± 1.1) *a	14.4 (± 1.3) *ab	5.381	*	0.002	5.129	**	0.034
INL	12.2 (± 0.6)	12.0 (± 0.6)	12.0 (± 0.7)	12.0 (± 0.5)	0.617	ns	0.6	0.037	ns	0.85
HL	8.4 (± 0.4) *a	8.2 (± 0.3) *b	8.2 (± 0.6) *ab	8.2 (± 0.4) *b	3.096	*	0.03	2.316	ns	0.13
HW	6.3 (± 0.4)	6.3 (± 0.3)	6.2 (± 0.5)	6.3 (± 0.4)	0.266	ns	0.85	1.512	ns	0.22
HH	4.5 (± 0.3) *a	4.5 (± 0.2) *ab	4.5 (± 0.3) *a	4.3 (± 0.2) *b	3.416	*	0.02	0.13	ns	0.72
HUM	4.8 (± 0.4)	4.7 (± 0.3)	4.6 (± 0.3)	4.8 (± 0.5)	1.088	ns	0.36	1.627	ns	0.21
RAD	4.5 (± 0.3)	4.5 (± 0.3)	4.5 (± 0.3)	4.4 (± 0.3)	0.444	ns	0.72	0.118	ns	0.73
HAL	6.9 (± 0.4)	6.8 (± 0.3)	6.7 (± 0.4)	6.8 (± 0.3)	1.063	ns	0.37	0.475	ns	0.5
FEM	6.4 (± 0.5)	6.2 (± 0.4)	6.3 (± 0.6)	6.6 (± 0.6)	1.735	ns	0.17	0.242	ns	0.624
TIB	7.4 (± 0.5)	7.4 (± 0.42)	7.4 (± 0.3)	7.5 (± 0.5)	0.554	ns	0.65	0.19	ns	0.66
FTL	12.6 (± 0.6)	12.2 (± 0.7)	12.2 (± 0.6)	12.3 (± 0.6)	2.322	ns	0.08	0.008	ns	0.93

*BL* body length, *FEM* femoral length, *FTL* foot length, *HH* head height, *HL* head length, *HUM* humeral length, *HW* head width, *INL* interbrachial-nasal length, *MAS* newborn mass, *RAD* radial (forearm) length, *SLV* snout-vent length, *TIB* tibial (foreleg) length, *TLL* tail length, *TOD* time of development, *TTL* total length. Significant effect of incubation temperature is indicated by (*) and significant effect on sex indicated by (**); letters *a, *b, & *c denotes the post-hoc difference among treatments; letters **m & **f denotes the post-hoc difference among sex; F, ANCOVA result; *ns* not significant; *p*, ANCOVA significance.

### Influence of incubation temperature and age on thermal physiology

**Figure 3:**
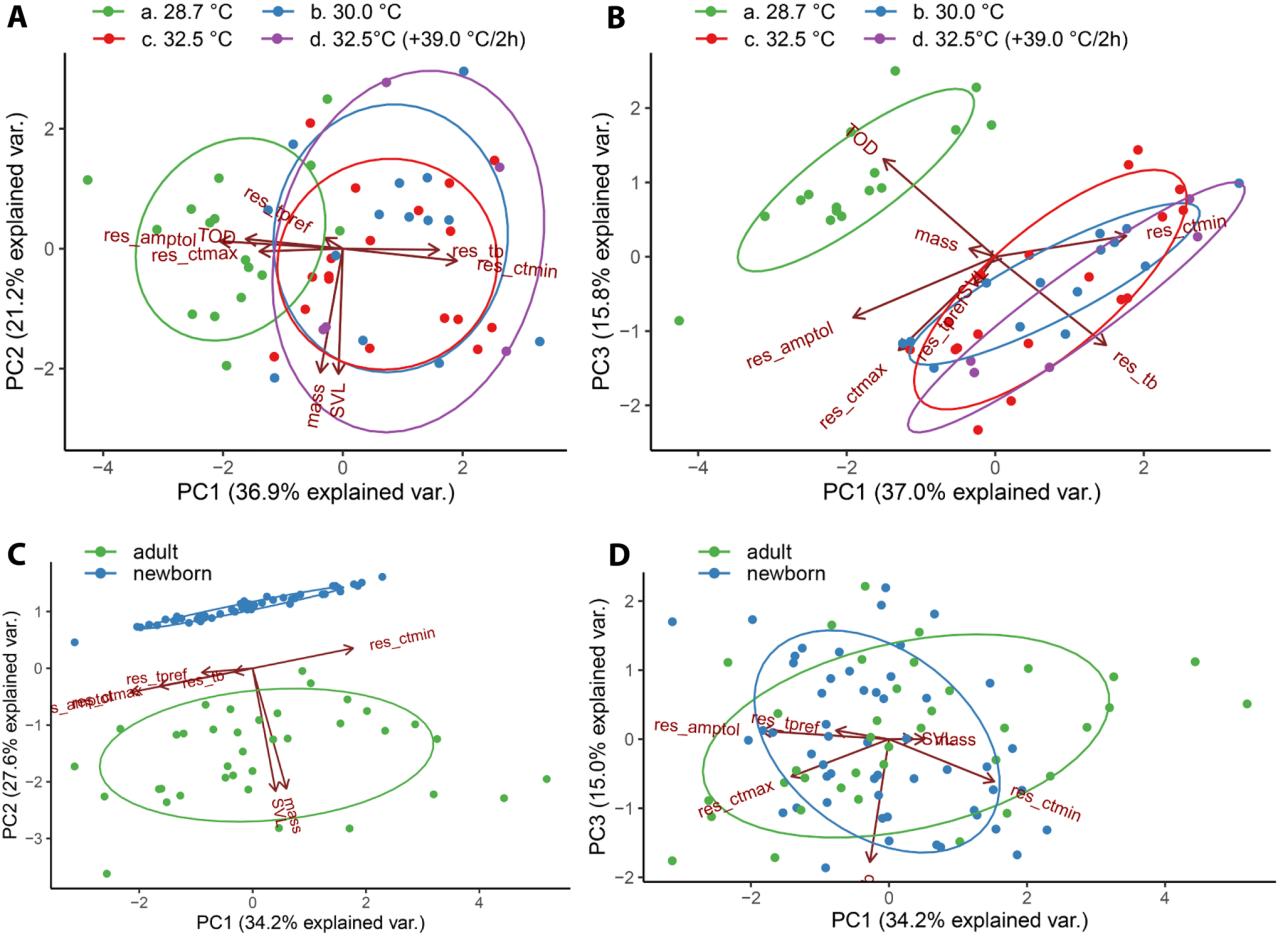
Principal component analysis biplot for incubation temperature and age effects over thermal physiological traits of *Tropidurus torquatus.* A-B, variation in incubation temperature effect at PC1 versus PC2 (**A**) and PC1 versus PC3 (**B**). C–D, variation in and age effect at PC1 versus PC2 (**C**) and PC1 versus PC3 (**D**). Abbreviations: explained var., explained variance; Mass, body mass; res_amptol, thermal-tolerance range residuals; res_ctmax, critical thermal maximum residuals; res_ctmin, critical thermal minimum residuals; res_tb, basal body temperature residuals; SVL, Snout-vent length; TOD, time of development (days).

Incubation temperature affected thermal traits (MANCOVA, Pillai = 0.744, F = 2.77, *P* < 0.001). ANCOVA tests showed significant differences in T_bb_ and CT_min_ traits for newborns incubated under artificial nest conditions showing lower means (Table 4). No significant differences were found in T_pref_, CT_max_ or Amp_tol_ values among the incubation temperatures (Table 4). T_pref_ was the only thermal physiological trait significantly affected by sex as a covariate at 30 °C treatment, with males showing higher preferred temperature than females (37.8 and 34.9 °C, respectively; ANCOVA, F = 4.685, *P* = 0.03). Our first PCA with TOD, SVL and mass as proxies for development and size, respectively, resulted in three PCs explaining 73.9% of the total variation (Table 6). PC1 accounted for 36.8% of the total variation and had a fair positive loading from CT_min_ (0.49), a fair negative loading from TOD (−0.41), a fair positive loading from T_bb_ (0.41), and a fair negative loading from Amp_tol_ (−0.53; Fig. 3A). This indicates a gradient from newborns with longer developmental periods tending to lower T_bb_ and CT_min_ values and higher thermal tolerance range. PC2 explained 21.2% of the total variation showing had very good negative loading from size parameters SLV (−0.71) and mass (-0.70), and did not show interpretable overlapping variance for thermal traits loadings. PC3 explained 15.9% of the total variation and had good positive loading from TOD (0.55), a fair negative loading from T_bb_ (−0.50), and a fair negative loading from CT_max_ (−0.53). This indicates a gradient from newborns with shorter developmental periods tending to higher CT_max_ and T_bb_ temperature values (Table 6; Fig. 3A,B, Supplementary Fig. SF3.A–E).

**Table 4:** Incubation temperature effects on thermal physiological traits of *Tropidurus torquatus* newborns under four incubation temperature regimes.

Trait	Incubation temperature (°C)				Effects					
	28.7 (n = 16)	30.0 (n = 14)	32.5 (n = 18)	32.5 (+ 39.0/2 h) (n = 5)	Incubation temperature			Sex		
					F statistics		*p*	F statistics		*p*
T_pref_	35.6 (± 2.2)	35.7 (± 2.5) **m > f	35.5 (± 2.1)	36.4 (± 1.5)	0.240	ns	0.86	4.865	**	0.03
T_bb_	25.6 (± 1.0) *b	28.5 (± 0.8) *a	28.3 (± 1.3) *a	29.0 (± 0.9) *a	26.884	*	< 0.0001	0.010	ns	0.92
CT_min_	11.6 (± 0.9) *b	13.4 (± 2.1) *a	13.4 (± 1.6) *a	13.8 (± 1.4) *a	4.894	*	0.005	0.347	ns	0.56
CT_max_	43.5 (± 1.4)	43.3 (± 1.3)	43.2 (± 12.)	43.3 (± 1.6)	0.179	ns	0.90	0.04	ns	0.84
Amp_tol_	31.9 (± 1.9)	29.8 (± 2.6)	29.7 (± 2.5)	29.5 (± 2.9)	0.279	ns	0.051	0.142	ns	0.71

*Amp*_*tol*_ thermal-tolerance range, *CT*_*max*_ critical thermal maximum, *CT*_*min*_ critical thermal minimum, *T*_*bb*_ basal body temperature, and *T*_*pref*_ preferred temperature. Significant effect of incubation temperature is indicated by (*) and significant effect on sex indicated by (**); letters *a & *b denotes the post-hoc difference among treatments; letters **m & **f denotes the post-hoc difference among sex; F, ANCOVA result; *ns* not significant; *p*, ANCOVA significance.

Thermal traits also differed significantly between newborns and adults for all analyzed traits, except for Amp_tol_ values (Table 5). Although newborns presented lower T_pref_ mean and larger T_pref_ variance that were significantly different from adults (Levene test, F = 5.26, *P* = 0.02), they proved to be less resistant to critical temperatures in terms of total means and also exhibited a small variance for CT_min_ (Levene, F = 12.6, *P* < 0.001) and CT_max_ (Levene, F = 10.921, *P* = 0.001). No difference was found in variance between newborns and adults for T_bb_ (Levene, F = 0.053, *P* = 0.81). Our second PCA with SVL and mass as proxies for body size/age resulted in three PCs explaining 76.8% of the total variation (Table 6). PC1 explained 34.2% of total variance concentrating thermal trait loadings. It had fair positive loading from CT_min_ (0.51), fair negative loading from CT_max_ (−0.47), and good positive loadings from Amp_tol_ (−0.62). PC2 explained 27.6% of the total variation and concentrates size traits loadings with very good negative loadings from SVL (−0.70) and mass (−0.68). PC3, with 34.2% of explained variance, concentrates the T_bb_ variance with excellent negative loadings (−0.90; Table 6; Fig. 3C,D).

**Table 5:** Age effect on the thermal physiological traits of *Tropidurus torquatus*.

Trait	Adults (n = 37)	Newborns (n = 53)	Age effect	
			F statistics	P
T_pref_	37.1 (± 1.2)	35.7 (± 2.1)	12.937	< 0.001
T_bb_	26.0 (± 1.5)	27.6 (± 1.7)	21.302	< 0.001
CT_min_	10.0 (± 2.6)	12.9 (± 1.8)	42.453	< 0.001
CT_max_	41.2 (± 2.2)	43.3 (± 1.3)	32.319	< 0.001
Amp_tol_	31.2 (± 3.8)	30.4 (± 2.5)	0.9405	0.33

*Amp*_*tol*_ thermal-tolerance range, *CT*_*max*_ critical thermal maximum, *CT*_*min*_ critical thermal minimum, *T*_*bb*_ basal body temperature, and *T*_*pref*_ preferred temperature. F, ANOVA result; P, ANOVA significance.

**Table 6:** Factor loadings of Principal Component Analysis of thermal and proxy-state related traits (A) incubation temperature and (B) age.

A			
Trait	PC1 (36.8% exp.var.)	PC2 (21.2% exp. var.)	PC3 (15.9% exp. var.)
SVL	−0.02	−0.71	−0.03
Mass	−0.10	−0.70	0.04
TOD	−0.41	0.06	0.55
res_tb	0.41	−0.01	−0.50
res_tpref	−0.07	−0.06	−0.14
res_ctmin	0.49	−0.07	0.11
res_ctmax	−0.36	−0.01	−0.53
res_amptol	−0.53	0.04	−0.35

B			
Trait	PC1 (34.2% exp.var.)	PC2 (27.6% exp. var.)	PC3 (15.0% exp. var.)
SVL	0.12	−0.70	0.002
Mass	0.17	−0.68	0.0001
res_tb	−0.09	−0.02	−0.90
res_tpref	−0.26	−0.02	0.06
res_ctmin	0.51	0.11	−0.31
res_ctmax	−0.47	−0.10	−0.27
res_amptol	−0.62	−0.14	0.06

*exp. var.* explained variance, Mass, body mass, *res_amptol* thermal-tolerance range residuals, *res_ctmax*, critical thermal maximum residuals, *res_ctmin* critical thermal minimum residuals, *res_tb* basal body temperature residuals, *SVL* Snout-vent length, *TOD* time of development (days).

## Discussion

The number of thermal developmental plasticity studies in squamate reptiles remains insufficient regarding the diversity of this group, which comprises one of the largest vertebrate radiations^4,41,46^. Moreover, there is a lack of in-depth studies on the effects of phenotypic plasticity drivers, such as temperature, during squamate development, particularly Neotropical lizards. Our study investigated the effect of incubation temperature on egg development, neonatal morphology, and thermal physiology of the tropidurid lizard *Tropidurus torquatus*, an emerging Neotropical model organism. In addition, our comparison of natural incubation regime and different artificial temperature regimes aimed to shed some light on the possible implications of climate change on the biology of this oviparous lizard. It is important to point out that the phenotypic characterization of *T. torquatus* presented in this work is based on different scenarios, where both incubation temperature regimes, constant temperatures—30.0, 32.5.0 and 35.0 °C—and floating temperatures—artificial nest and 32.5 °C + 39.0 °C/2 h/day—were considered. A growing number of recent studies have sought to understand the dynamics of constant and fluctuating incubation temperature scenarios on embryo survival and newborn fitness^5,47–49^. It is reasonable that those conducted only with constant temperature regimes are considered to be ecologically less relevant, even though it is of fundamental importance to understand the dynamics of embryonic development of oviparous reptiles^50,51^.

Egg mass and volume showed significant variation during development, both of which were positively affected by incubation temperature. Mass gain reached an increase of up to four times the egg mass at the time of oviposition. However, this difference was not mirrored in the mass of hatched newborns, which was neither affected by incubation temperature nor egg mass gain. Previous studies^23,26^ argued that egg mass gain during the incubation period does not influence embryo development, even if results show that egg mass gain by water absorption from the surrounding environment is affected by temperature. Our finding corroborates those studies, as there was no difference in neonate mass between incubation temperatures at the time of hatching, even though egg mass showed a positive temporal water gain. Nonetheless, this phenomenon should be investigated in the future, not only from the perspective of the direct influence of water absorption on mass gain, but also regarding the degree of thermoregulatory control due to the absorbed water inside the eggs^52,53^.

In nature, oviparous species generally tend to choose locations that best mitigate the effect of environmental variables on egg development^54,55^. In addition to suitable nesting site choice, most oviparous squamate species have a flexible eggshell enabling water uptake from the laying environment, taking advantage of the combined action of shell porosity and yolk osmolarity^56^. This characteristic likely provides thermal and mechanical protection to the embryo due to the properties of the aqueous layer formed in the space between the embryo and the shell. It is reasonable to hypothesize that this characteristic doubly benefits the embryo by: (i) increasing egg resistance against mechanical pressures, and (ii) protecting the embryo from abrupt temperature variations, as the thermodynamic properties of water in this layer promotes thermal insulation and an environment favorable to thermal equilibrium. Temperature-induced egg mass and volume gains have been documented for other squamate species with flexible eggshells, a characteristic that has possibly contributed to their developmental success^57,58^. Nevertheless, not all squamates have flexible eggshells capable of acting as external incubators during post-ovipositional development, as is likely the case in *T. torquatus* and other species with flexible eggshells. An unanswered question remains regarding the influence of this phenomenon on embryo survival rates, also concerning species that have rigid calcareous eggshells.

While some squamate species experience a more stable incubation temperature regime due to viviparity, the vast majority of squamates are oviparous, whose embryonic development is directly affected by factors of the egg laying environment, especially temperature and humidity^6,59,60^. As in several oviparous reptile species studied^23^, the incubation temperature negatively affected the survival rate and incubation period of *Tropidurus torquatus*. This general pattern, also reported in other lizard species, is influenced by several factors such as environmental adaptation, and the phylogenetic component itself^23,61–65^. It is noteworthy that *T. torquatus* exhibits a low tolerance to increased incubation temperature, with 100% lethality observed for eggs incubated at 35.0 °C. This incubation temperature still generates viable neonates in other species^66^, albeit with a considerably lower birth rate.

The sex ratio of *Tropidurus torquatus* proved to be remarkably susceptible to genetic-environment interaction in a certain incubation temperature range. While artificial nest and 32.5 °C ± 39.0 °C/2 h treatments had a 1:1 sex ratio, the 30.0 °C treatment had a statistically significant higher proportion of females, while the 32.5 °C treatment did not have a statistically higher proportion of males. However, the statistically significant mixed proportion with a greater number of females recorded in the 30.0 °C treatment group only allows us to hypothesize that sexual determination in *T. torquatus* can occur by an interaction between environmental and genetic factors, but that this is not a species with temperature-dependent sexual determination as there was no temperature range that registered an exclusivity of male or female neonates. In species where the interaction between environmental and genetic factors has been demonstrated, the duration and magnitude of the exposure of eggs to more masculinizing/feminizing temperatures acts over hormonal expression processes that ultimately determine the development of either the testes or ovaries^4^. In addition to being the only treatment with a significant sex ratio difference, neonates hatched at 30 °C were the only ones to show a significant difference in T_pref,_ with male newborns showing higher preferred temperatures. Future studies may clarify the possible existence of sex reversal mechanism in the species or a possible susceptibility of male embryos in this temperature range^29,30^.

Recent studies revealed that changes in the morphology, growth and sexual maturity and decline of *Tropidurus torquatus* populations have accompanied climatic changes in the last decades^18,67^. Our data shows that incubation temperature has an effect over body size traits. While the body and tail of *T. torquatus* are positively affected by increased incubation temperature, the size and height of the head are negatively affected. Not surprisingly, several studies have shown that the incubation temperature affects morphological traits, however, the ecological and physiological consequences of these changes remain unknown. In fact, there is a possibility that an increase in body size will benefit the species since reproductive characteristics such as litter size are evolutionarily related to body size. On the other hand, a larger body demands greater energy gain. This goes against the prediction that lizard populations will be negatively affected by global warming, since there will be a decrease in the potential daily foraging period, thus affecting energy gain^21^.

Despite the substantial influence of thermal physiology on the survival of neonates in the initial periods of post-hatch life, this topic remains insufficiently explored^41,68,69^. Few studies have been conducted with the aim of understanding the effects of incubation temperature on the thermal physiology of newborn squamates, still, comparisons across taxa show that the influence of incubation temperature on thermal traits exhibit conflicting patterns^70–72^. As in other lizard species studied, there was no change in the selected average temperatures of *T. torquatus* newborns hatched under different incubation regimes^52^. However, higher incubation temperatures significantly increased the metabolic rates of embryos and neonates, which was reflected in the reduced incubation period and high T_bb_ values positively affected by incubation temperature in *T. torquatus*. As expected, incubation temperature is a factor promoting a compensatory relationship between developmental time, metabolic rate, and energy expenditure during embryonic development^73,74^. This also extends into the early neonatal stages of life, as reflected in T_bb_.

The influence of incubation temperature on the critical thermal maximum and minimum of *T. torquatus* newborns may render this species more vulnerable to future predicted warming: resistance to critical cold temperature values by neonates decreases significantly with incubation temperature increments. In fact, *T. torquatus* shows plasticity in CT_min_ values with negative implications since the neonatal CT_min_ values increase at higher incubation temperatures. This reduction in CT_min_ renders the species more vulnerable to abruptly colder climatic events, whose occurrence frequency is predicted to increase in the climate change scenarios. On the other hand, the absence of plasticity in CT_max_ values indicates that this trait is possibly in a limited state of variation. The fact that incubation temperature does affect the CT_max_ values of *T. torquatus* should make this species more vulnerable to temperature changes in foraging environments induced by global warming. If unable to adapt to the predicted increase in maximum daily temperature, the daily activity period of *T. torquatus* dedicated to foraging may indeed be circumstantially reduced, impacting important biological aspects such as energy gain and mate searching^71^.

Few studies have investigated the effect of age on the thermal physiology of squamate reptile neonates. The variation in critical thermal maximum and minimum in the few species investigated shows that there is no general rule regarding whether neonates are more or less susceptible to higher environmental temperatures than adults. Adults of the lizard species: *Eremias multiocellata* (Lacertidae), *Stenocercus guentheri* (Tropiduridae), and *Podarcis siculus* (Lacertidae) showed higher CT_max_ values ​​than juveniles, which are more tolerant than adults in the *Sceloporus jarrovii* (Phrynosomatidae) lizard and the *Nerodia rhombifer* (Colubridae) snake^68^. Cases where there is no difference in the CT_max_ between juveniles and adults include the lizard species: *Aspidoscelis sexlineatus* (Teiidae) and *Oligosoma maccannii* (Scincidae)^68^. A similar degree of variation across taxonomic groups has also been observed for the CT_min_. The pattern observed from the thermal physiological traits of *T. torquatus* indicates that newborns thermoregulate in a lower temperature range compared to adults. In addition, newborns have less resistance to colder temperatures and greater resistance to higher temperatures than adults.

In conclusion, our study provides an in-depth investigation of the effects of incubation temperature on the plasticity of development, morphology, and thermal physiology of the emerging Neotropical lizard model species *T. torquatus*. Furthermore, it is the first study to investigate such effects in a lizard species of the Brazilian Cerrado. Anthropogenic actions have increased the susceptibility of this biome to ensuring its biodiversity, as well as its ecosystem services. The vast fragmentation makes it increasingly susceptible to environmental variations caused by global warming, directly affecting the phenotype and stability of populations. Our experimental findings suggest that modifications in incubation temperatures due to an increase in nest site temperatures caused by global warming may affect several features of the natural history, morphology, and thermal physiology of newborns of the collared lizard. In addition, our results highlight the importance of observing such traits from the point of view of the global climate change paradigm and its implications for the conservation of lizard diversity.

## Methods

A total of 144 eggs were collected from 25 pregnant adult females of the *Tropidurus torquatus* species during the breeding season (Oct/2018–Jan/2019) from urban populations of Brasília (15°45′46.79″ S, 47°52′05.34″ W), Distrito Federal, Brazil. The females were collected by noosing and kept in terrariums with medium grain vermiculite/washed sand (1:1) as a substrate, with a 12/12 h light/dark cycle until oviposition. In order to minimize distress, four or five females were placed in 60 × 40﻿ × 50 cm terrariums supplied with food (live cockroaches *Nauphoeta cinerea* floured with calcium and vitamin supplements for reptiles), fresh water and heating plates at 35 °C for thermoregulation. Animal capture was licensed by the Brazilian Ministry of the Environment (ICMBio/SISBIO permit n° 63226-1). The experimental procedure described herein was fully approved and performed in accordance with the University of Brasilia Ethics Committee (CEUA-UnB permit n° 116/2017). Also, the experimental design is in accordance with the ARRIVE guidelines 2.0^75^.

Terrariums were inspected daily for the presence of eggs. Once oviposition occurred, the eggs were individually identified and their mass, length and width determined with aid of a precision digital balance and calipers, respectively. Each egg was transferred to a 50 mL container with 10 g vermiculite and 20 mL water, conferring a saturated humidity environment. Finally, the containers were labeled and randomly distributed in five treatment groups: three egg incubators set at constant temperatures: (i) 30.0 °C (n = 33), (ii) 32.5 °C (n = 28), (iii) 35 °C (n = 22); (iv) an egg incubator set at 32.5 °C with variation to a peak temperature of 39.0 °C for 2 h per day (n = 31), and (v) an artificial nest (n = 30) built in the field.

The construction of the artificial nest was based on our observations of the behavior of some females that spawn at wall corners under concrete slabs. Thus, the artificial nest consisted of a wire cage placed at an external wall corner covered with concrete slabs equipped with a datalogger for temperature monitoring at 10-min intervals. The mean temperature of the artificial nest treatment (28.7 °C) was calculated based on the mean of 20,069 readings. Artificial nest temperature fluctuations were more pronounced at the maximum daily temperatures, as the estimated mean temperature was 0.7 °C above the median temperature registered (28.0 °C). The mean maximum daily temperature was 39.1 °C, while the mean daily minimum temperature was 22.7 °C. The maximum and minimum temperatures registered were 45.8 °C and 20.9 °C, respectively (Supplementary Fig. SF1).

Each treatment was inspected daily for newborns. Egg measurements (mass, length and width) were repeated at 15-day intervals in order to minimize egg handling and embryo distress. Once an egg hatched, the newborn was immediately weighed, placed in a plastic container and transferred to the laboratory for acclimation at room temperature (~ 25.5 °C) for 24–48 h, prior to the thermal experiments. After the experiments, newborns were euthanized with an intraperitoneal injection (0.1 mL of 2% lidocaine hydrochloride), fixed in 10% formalin and preserved in 70% ethanol. The specimens were deposited in the collection of the Laboratory of Vertebrate Comparative Anatomy–Department of Physiological Sciences–University of Brasília (LACV–CFS–UnB).

### Developmental traits

Incubation and egg development were evaluated with respect to time of development (TOD), egg mass and volume. Egg volume was calculated using the ellipsoid formula: $v=\frac{4}{3}\pi a{b}^{2}$, in which $a$ corresponds to the major radius ($\frac{1}{2}$ egg length) and $b$ corresponds to the minor radius ($\frac{1}{2}$ egg width). Final egg mass was estimated as the mean of the last measurement taken from eggs before hatching.

### Morphological variables

The morphological dataset comprises data on sex, mass, and 15 body measurements from 82 newborns: SVL—snout-vent length; TLL—tail length; TTL—total length; BL—body length (pelvic girdle-neck fold length); INL—interbrachial-nasal length; HL—head length; HW—head width; HH—head height; HUM—humeral length; RAD—forearm length; HAL—hand length; FEM—femoral length; TIB—foreleg length, and FTL—foot length. For sex determination, the specimens were dissected under a stereoscopic microscope for examination of gonads and presence of oviduct primordia. Mass and morphological variables were measured with the aid of a digital balance and calipers, respectively.

### Thermal physiology experiments

We collected thermal physiology data from 53 lab-incubated newborns and 37 adults of *Tropidurus torquatus* (18 females, 73–104 mm/SVL and 19 males, 88–112 mm/SVL). Adult lizards come from the same collection site as the pregnant females (Brasília, Distrito Federal, Brazil, 15°45′46.79″ S, 47°52′05.34″ W). Five thermal traits were measured: basal body temperature (T_bb_), preferred temperature (T_pref_), critical thermal minimum (CT_min_), critical thermal maximum (CT_max_), and the thermal-tolerance range (Amp_tol_). All measurements were based on cloacal temperature and recorded using a 1 mm diameter Type K thermocouple probe inserted 5 mm (newborns) to 10 mm (adults) into the cloaca.

Basal body temperature (T_bb_) was determined as the 24–48 h post-acclimation temperature. The preferred temperature (T_pref_) experiment consisted of placing each specimen in a thermal gradient with air temperature ranging from 10 to 40 °C. This temperature gradient was built using a halogen lamp (100 W) and an ice pack placed at opposite sides of a 1 m-length glass container wrapped with black self-adhesive contact paper to avoid specimen visual distraction. The final preferred temperature was estimated as the mean of all body temperature values taken every 10–20 min for 2 h. The critical thermal minimum (CT_min_) was evaluated by placing the specimen in a cold chamber with an air temperature of ~ 4 °C provided by ice packs positioned 4 cm beneath the substrate, avoiding direct contact of the specimen with the ice pack. The chamber was cooled at a constant rate of −2.0 °C/min. For critical thermal maximum (CT_max_) evaluation, the specimen was placed in a hot chamber with heating provided by a constant input of hot water steam. The chamber was heated at a constant rate of 4.0 °C/min. In both cases, the critical minimum and maximum body temperatures corresponded to the temperatures at which the specimen lost its righting response. The thermal-tolerance range (Amp_tol_) is the difference between the critical thermal maximum and minimum temperatures. Experiments were carried out at an interval of 24 h to avoid interference. Both neonates and adults were measures within the same time period (Nov/2018–May/2019), under the same experimental parameters and equipment. The experiments were carried out in the afternoon for all individuals, always startin at 2:00 pm.

### Data analysis

All analyses and graphics were performed using the R statistical platform version 3.6.2^76^. Normality and homoscedasticity were verified on log-transformed data prior to analysis. Variation in egg mass and volume during the developmental period were analyzed with ANCOVA, using the incubation period as a covariate. We also tested for mass differences between eggs and newborns using ANOVA. Final egg mass was estimated by the last egg measurement taken during the incubation period. General estimates of incubation temperature effects on morphology and thermal physiology were tested with MANCOVA. Differences in mortality rate and sex ratio among treatments were tested using the Pearson’s Chi-squared test applying Yates correction for small sample size. Finally, morphological traits were analyzed by linear regressions and ANCOVAs, setting SVL and sex as covariates. For thermal physiological traits, we also performed linear regressions and ANCOVAs, with SVL and sex as covariates. Latent relationships in the data were identified by performing Principal Component Analysis (PCA) for incubation temperature and age effects. After performing the analysis, factor loadings were interpreted based on Tabachnik and Fidell^77^ criteria for overlapping variance classification: excellent (> 0.71), very good (0.7 ≤ 0.63), good (0.62 ≤ 0.55), fair (0.54 ≤ 0.45), poor (0.44 ≤ 0.32) or uninterpretable (< 0.32).

## Supplementary Information

Supplementary Information.

## Data Availability

The datasets generated during and/or analyzed during the current study are available from the corresponding author on reasonable request.
