# Supplementary material for: Effects of incubation temperature on development, morphology, and thermal physiology of the emerging Neotropical lizard model organism Tropidurus torquatus
Source: Sci Rep. 2022 Oct 13;12:17153. doi: 10.1038/s41598-022-21450-7 (PMC9562357; doi:10.1038/s41598-022-21450-7)
Supplement: Supplementary file 1 — Supplementary Information. [file 41598_2022_21450_MOESM1_ESM.pdf]

Supplementary Information

Effects of incubation temperature on development, morphology, and thermal physiology of the emerging Neotropical lizard model organism *Tropidurus torquatus*

Anderson Kennedy Soares De-Lima[1]*, Carlos Henke de Oliveira[1], Aline Pic-Taylor[1] & Julia Klaczko[1,2] *

[1]University of Brasília, Brasília, DF, 70910-900, Brazil

[2]Department of Life Sciences, Natural History Museum, London SW7 5BD, UK

*Corresponding author (aksdelima@gmail.com; jklaczko@unb.br)

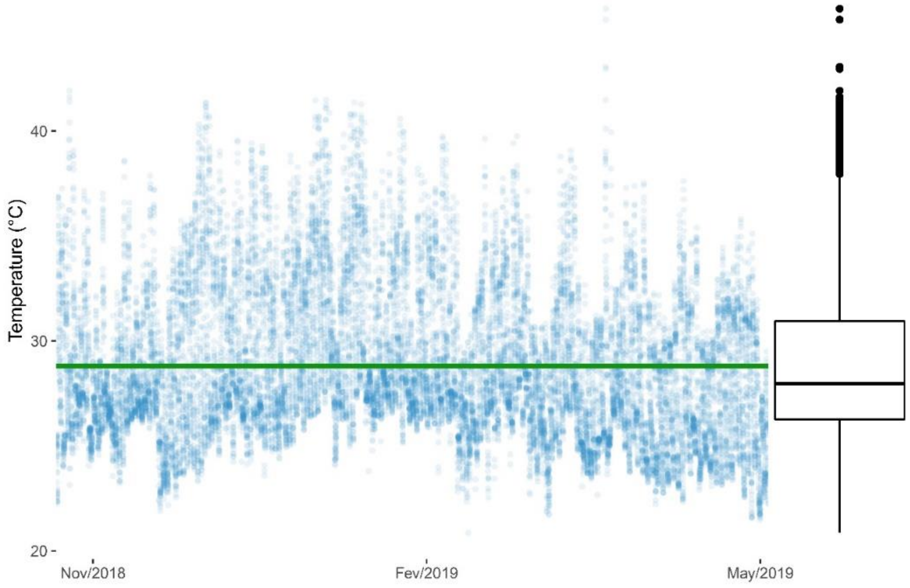

Supplementary figure SF1. Variation in artificial nest temperature during incubation period of *Tropidurus torquatus* eggs (Nov/2018 - May/2019)

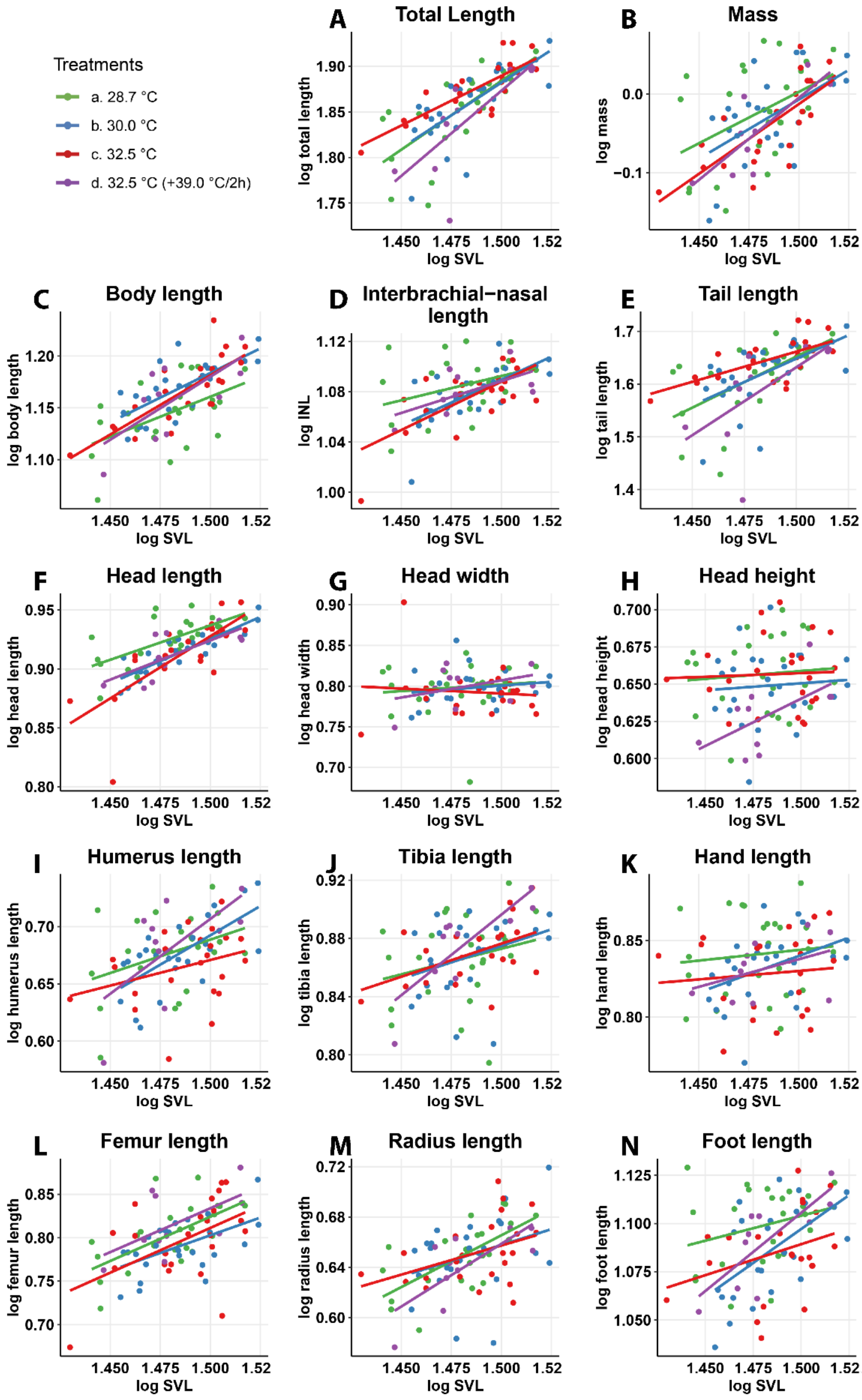

Supplementary figure SF2. Variation in morphological traits of *Tropidurus torquatus* newborns hatched under four different incubation regimes. Abbreviations: INL, Interbrachial-nasal length; SVL, Snout-vent length.

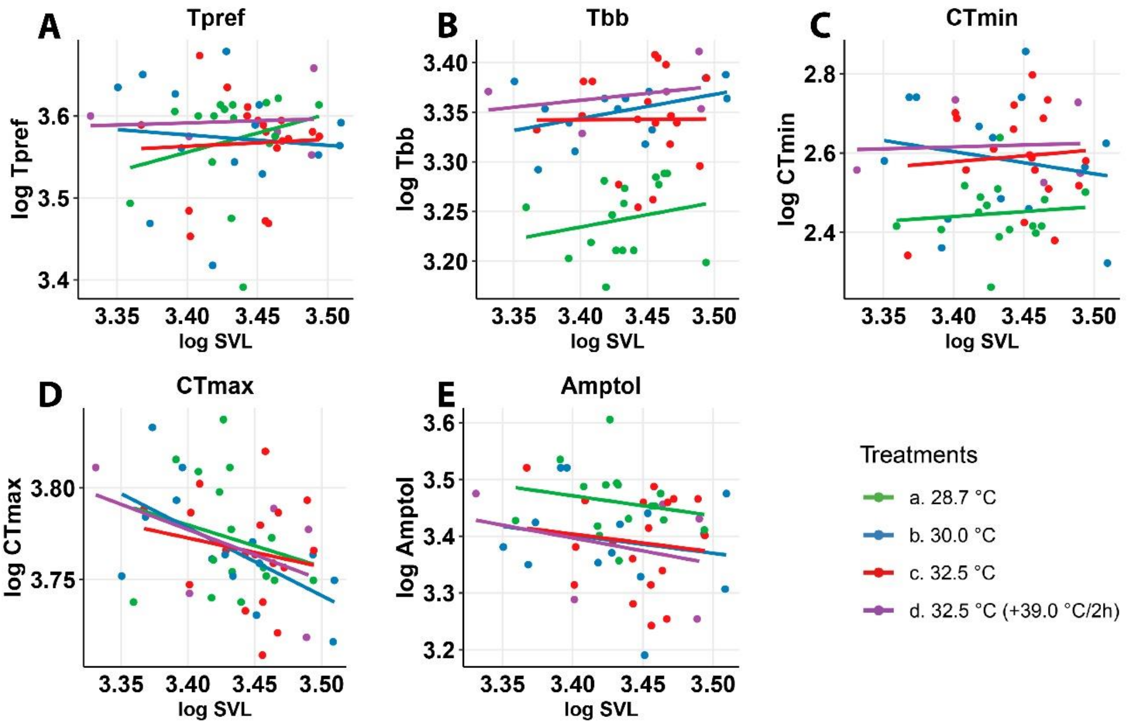

Supplementary figure SF3. Variation in thermal physiology traits of *Tropidurus torquatus* newborns hatched under four different incubation regimes. Abbreviations: Amp_{tol}, thermal-tolerance range; CT_{max}, critical thermal maximum; CT_{min}, critical thermal minimum; T_{bb}, basal body temperature, and T_{pref}, preferred temperature. SVL, Snout-vent length.
